# Locked twins—remote from term: a case report

**DOI:** 10.1186/s13256-021-02725-5

**Published:** 2021-03-12

**Authors:** Abraham Fessehaye, Ferid A. Abubeker, Mekdes Daba

**Affiliations:** Department of Obstetrics and Gynecology, Saint Paul’s Hospital Millennium Medical College, Addis Ababa, Ethiopia

**Keywords:** Twin pregnancy, Locked twins, Low-vertical cesarean section, Breech

## Abstract

**Background:**

Locked twins is a rare and hazardous obstetric complication, which occurs in approximately 1:1000 twin pregnancies. One of the known etiologic factors for locked twins is size of the twins. We report a case of chin-to-chin locked twins that occurred at gestational age of 30 weeks pus 6 days.

**Case summary:**

A 27 years-old primigravida Ethiopian mother presented with a history of pushing down pain and passage of liquor of 6 hours duration at gestational age of 30 weeks plus 6 days. With a diagnosis of twin pregnancy (first twin non-vertex), abdominal delivery was decided in latent first stage of labor but mother refused caesarian delivery and she was allowed to labor with the hope of achieving a vaginal delivery. In second stage, interlocking twin was encountered and a low vertical cesarean section was done to effect delivery of twins without the need to decapitate the first twin.

**Conclusion:**

Locked twin is a rare obstetric complication. Whenever it is encountered, successful delivery can be achieved without the need to have decapitation of the first twin during caesarian section.

## Background

Of the different etiological factors for locked twins, the most important are the age of the mother, parity of the mother, and the size of the twins [1]. Interlocking twins are more common in primigravida as basal uterine tone may be greater in the first pregnancy resulting in stronger uterine contractions during labor [2]. This report describes a 27 years-old primigravida mother who complicated with a chin-to-chin locked twins at early 3rd trimester of pregnancy, at gestational age of 30 weeks and 6 days. Low vertical cesarean section was done that successfully effected delivery of the babies without any need of decapitation of the first twin. This is a unique surgical practice being reported in our case for the first time.

## Case presentation

A 27 years-old primigravida Ethiopian mother presented with a history of pushing down pain and passage of liquor of 6 hours duration at gestational age of 30 weeks plus 6 days from a 24 weeks ultrasound. Her last normal menstrual period was unknown. She had no history of vaginal bleeding. She had no history of fever. She had no history of other underlying obstetric or medical problem. She had antenatal care follow-up at our hospital. Her medical, surgical, psychosocial, and family history was unremarkable. Obstetric ultrasound was done twice in the second trimester (at 24 weeks and 26 weeks gestation) which documented a normal dichorionic-diamnotic (DCDA) twin pregnancy with no evidence of any congenital anomaly.

Up on physical examination, the vital sign of the patient was stable. Her blood pressure was 110/70 mmHg. She had a pulse rate of 88 beats per minute, temperature of 37 °C, and respiratory rate of 16 breaths per minute. The pertinent finding was on pelvic examination—cervix was 3 cm dilated with 80% effacement. First twin was in complete breech presentation at station of − 1 and membrane was ruptured and clear liquor.

With the assessment of latent phase of labor plus twin pregnancy (DCDA) first twin in breech presentation plus early preterm pregnancy, obstetric ultrasound was done. The sonographic findings was first twin in breech presentation with estimated fetal weights of Twin A of 1200 g and twin B cephalic presentation 1100 g. The biophysical profile for both twins was reassuring. Baseline fetal heartbeat was 126 and 140 for twin A and twin B, respectively. With an indication of twin pregnancy (first non-vertex) in labor, emergency cesarean section (CS) was decided but patient refused for the CS delivery despite being thoroughly counseled repeatedly.

The fetal heart rate for both twins was monitored using CTG at labor ward and it was normal throughout until she entered second stage of labor. She entered second stage of labor after 4 hours of labor. One hour in to the second stage, at station of + 2, locked twin was diagnosed. By that time, first twin was delivered up to the shoulders. Consultant on duty was consulted immediately, and manual maneuvers was attempted to dislodge the interlocking. However, it was not successful multiple times. Still, it was difficult to convince the mother to undergo abdominal delivery after 5 minutes of trial of the maneuvers. By the time our patient agreed for CS delivery, the first baby was already asphyxiated and fetal heart beat was negative when we arrived at the operation theater. The fetal heartbeat of the second twin was also detected to be in bradycardic range (106–112 beats per minute) as it was checked before proceeding with CS delivery.

With an indication of locked twins Cesarean delivery was done, a low-vertical CS. First, second twin in cephalic presentation was extracted after the chin-to-chin twin interlocking was relieved manually. The attending surgeon's right hand rested on the lateral side of the second baby's face and it slipped the chin of the second twin with a gently push from the lateral side. Then, the head of the second twin was extracted cephalic by a push technique.

Delivery of the babies was effected without the need of decapitation of the first twin. The outcome was a 1000 g female second twin born with APGAR score of 4, 2, and 2 in the first, 5th and 10 minutes respectively. The baby passed away after 20 minutes of resuscitation. Chest compressions synchronized with a positive pressure ventilation, and administration of adrenaline three doses was instilled. Subsequently, the first twin, a 1200 g male still-born was delivered vaginally with simple traction from below. The mother was counselled for autopsy of both neonates but she declined it. The decision to delivery time was 20 minutes. The mother was discharged with full recovery after three days of stay in the maternity ward. Her post-operative follow-up visit after a week didn’t document any abnormality. She had a good wound healing.

## Discussion

Twin pregnancies are at increased risk of intrapartum complications [1]. Locked twins is a rare, hazardous obstetric complication, which occurs in approximately 1:1000 twin pregnancies [2, 3]. Locked twins usually occur when the after-coming head of the first breech fetus is locked with the head of the second cephalic fetus. Of the different etiological factors, the most important are the age and parity of the mother and the size of the twins [2]. The perinatal mortality rate is in excess of 40 per cent. This is due mainly to difficulty in disengaging the two heads, with the consequent death of one or both twins [3].

What makes our case unique is that cesarean delivery (CS) was done without decapitation of the first twin. The stated difficulty of disengaging the two heads of the neonates was addressed by making a wide uterine incision—low vertical incision—that allowed for safe dislodging of the interlocking intra-operatively. The death both neonates in our case is explained by the significant delay to do CS delivery as the mother repeatedly refused to undergo for it.

Lawrence (1949) classified cases of locking into four groups according to the respective presentation of the twins. These are, in order of frequency, breech and vertex, vertex and vertex, vertex and transverse, and breech and breech. Nissen (1958) followed Lawrence's classification. In his collection a little more than two thirds belonged to the first group [4]. Our case is classified on the first group.

Interlocking above the pelvis does not present a dangerous outcome for both twins, and caesarean section may be favored. Interlocking at or below the pelvic brim, like it occurred in our case, is a serious complication. Usually, the diagnosis of locked twins is made in the second stage of labor. In this situation, vaginal delivery of both twins may be possible if the fetuses are small, the pelvis is roomy and congenital fetal anomalies are absent. The Kimball–Rand maneuver of hyperextension and traction of the first twin and flexion and traction of the second head, using Piper forceps, may result in the simultaneous delivery of both heads; otherwise, delivery of the second twin past the first by forceps may be performed [5]. The other option reported in the literature is the Zavanelli manoeuvre [6]. In our case, manual maneuver was tried to resolve the interlocking but it was not successful. Finally, low vertical CS was done.

If the diagnosis of locked twins is made after the death of first twin, the demised baby will be decapitated, pushed upward for a safe delivery of the second fetus [7]. Our case was managed without the need of decapitation of the first twin, which was achieved by creating adequate space for manipulation by using a low-vertical uterine incision.

## Conclusion

When locked twins occurs, cesarean section is the recommended mode of delivery. But, it should not be a hard and fast rule that extraction of the babies be achieved by decapitation of the first twin. If adequate space is created by using low vertical (like it was done in our case) or inverted T C-section, delivery of the twins can be effected without decapitation of the first twin.

## Availability of supporting data

All supporting documents are submitted along with the case report.

## References

[1] Goossens SMTA, Roumen FJME, Derks JB, Kessels FG, Dirksen CD, Nijhuis JG. Planning the mode of delivery for twin pregnancies: a web-based questionnaire. J Obstet Gynaecol. 2016;36(2):172–7. 10.3109/01443615.2015.1030730. 26367456

[2] Borah T, Das A. Locked twins: a rarity. Ann Med Health Sci Res. 2012;2(2):204. 10.4103/2141-9248.105676 PMC3573520 23439954

[3] Sevitz H, Merrell DA. The use of a beta-sympathomimetic drug in locked twins: case report. BJOG Int J Obstet Gynaecol. 1981;88(1):76–7. 10.1111/j.1471-0528.1981.tb00942.x 7459295

[4] Deiry SKEL. Two cases of locked twins. 1960;1174–7. 10.1136/bmj.1.5180.1174 PMC1967169 13819303

[5] Saad FA, Sharara HA. Locked twins: a successful outcome after applying the Zavanelli manoeuvre. J Obstet Gynaecol. 1997;17(4):366–7. 10.1080/01443619750112853 15511888

[6] Kerbage Y, Coulon C, Subtil D, Garabedian C. Locked twins: successful vaginal delivery of both twins after Zavanelli manoeuvre of Twin B. Eur J Obstet Gynecol Reprod Biol. 2016;207:236–7. 10.1016/j.ejogrb.2016.10.035 (**Epub 2016 Oct 28**). 27825768

[7] Renfrew MJ, McFadden A, Bastos MH, Campbell J, Channon AA, Cheung NF, Silva DR, Downe S, Kennedy HP, Malata A, McCormick F, Wick L, Declercq E. Midwifery and quality care: findings from a new evidence-informed framework for maternal and newborn care. Lancet. 2014;384(9948):1129–45. 10.1016/S0140-6736(14)60789-3. Erratum in: Lancet. 2014;384(9948):1098. **(Epub 2014 Jun 22)**. 24965816

