# Correction to: Locked twins—remote from term: a case report

**DOI:** 10.1186/s13256-021-03130-8

**Published:** 2021-10-08

**Authors:** Abraham Fessehaye, Ferid A. Abubeker, Mekdes Daba

**Affiliations:** Department of Obstetrics and Gynecology, Saint Paul’s Hospital Millennium Medical College, Addis Ababa, Ethiopia

## Correction to: J Med Case Rep (2021) 15:115 https://doi.org/10.1186/s13256-021-02725-5

Following publication of the original article [1], the authors would like to correct the approximate number of locked twins pregnancies in the Abstract under the heading Section "Background" and in the main article under the heading Section "Discussion".

The sentence currently reads:

Locked twins is a rare and hazardous obstetric complication, which occurs in approximately 1:100 twin pregnancies.

The sentence should read:

Locked twins is a rare and hazardous obstetric complication, which occurs in approximately 1:1000 twin pregnancies.

The sentence currenty reads:

Locked twins is a rare, hazardous obstetric complication, which occurs in approximately 1:100 twin pregnancies [2, 3].

The sentence should read:

Locked twins is a rare, hazardous obstetric complication, which occurs in approximately 1:1000 twin pregnancies [2, 3].

The original article [1] has been corrected.
